# Prognostic Value of a Quantitative Analysis of Lipoarabinomannan in Urine from Patients with HIV-Associated Tuberculosis

**DOI:** 10.1371/journal.pone.0103285

**Published:** 2014-07-30

**Authors:** Andrew D. Kerkhoff, Robin Wood, Monica Vogt, Stephen D. Lawn

**Affiliations:** 1 George Washington University School of Medicine and Health Sciences, Washington, DC, United States of America; 2 Desmond Tutu HIV Centre, Institute of Infectious Disease and Molecular Medicine, Faculty of Health Sciences, University of Cape Town, Cape Town, South Africa; 3 Department of Global Health and Amsterdam Institute for Global Health and Development, Academic Medical Center, University of Amsterdam, Amsterdam, the Netherlands; 4 Department of Clinical Research, Faculty of Infectious and Tropical Diseases, London School of Hygiene and Tropical Medicine, London, United Kingdom; 5 Department of Medicine, Faculty of Health Sciences, University of Cape Town, Cape Town, South Africa; Cambridge University, United Kingdom

## Abstract

**Background:**

Detection of the mycobacterial cell wall antigen lipoarabinomannan (LAM) in urine can be used to diagnose HIV-associated tuberculosis (TB) using a qualitative (positive/negative) read-out. However, it is not known whether the quantity of LAM present in urine provides additional prognostic information.

**Methods/Findings:**

Consecutively recruited adult outpatients initiating antiretroviral therapy (ART) in South Africa were investigated for TB regardless of clinical symptoms using sputum smear microscopy and liquid culture (reference standard). Urine samples were tested using the Clearview TB-ELISA for LAM and the Xpert MTB/RIF assay. The ELISA optical densities (OD) were used as a quantitative assessment of urine LAM. Among 514 patients with complete sputum and urine LAM OD results, culture-confirmed TB was diagnosed in 84 patients. Twenty-three (27.3%) were LAM-positive with a median LAM OD of 0.68 (IQR 0.16–2.43; range, 0.10–3.29) and 61 (72.6%) were LAM negative (LAM OD <0.1 above background). Higher LAM ODs were associated with a range of prognostic indices, including lower CD4 cell counts, lower haemoglobin levels, higher blood neutrophil counts and higher mycobacterial load as assessed using both sputum and urine samples. The median LAM OD among patients who died was more than 6.8-fold higher than that of patients who remained alive at 3 months (P<0.001). The small number of deaths, however, precluded adequate assessment of mortality risk stratified according to urine LAM OD.

**Conclusions:**

In patients with HIV-associated TB, concentrations of LAM in urine were strongly associated with a range of poor prognostic characteristics known to be associated with mortality risk. Urine LAM assays with a semi-quantitative (negative vs. low-positive vs. high-positive) read-out may have improved clinical utility over assays with a simple binary result.

## Introduction

Tuberculosis remains the leading cause of mortality among people living with HIV/AIDS (PLWHA) both in sub-Saharan Africa and globally [1]. This is in part due to the non-specific clinical presentation among HIV-infected patients such that much TB disease remains undiagnosed and therefore untreated. Diagnosis is further complicated by high rates of disseminated, extra-pulmonary, sputum smear-negative and radiologically non-specific disease [2]–[4]. Improved microbiological assays for the detection of TB among PLWHA that are accurate, low-cost and easy to implement at the point-of-care are therefore an important public health priority [5]–[7].

Lipoarabinomannan (LAM) is a cell wall antigen of *Mycobacterium tuberculosis* that can be detected in urine, providing utility for the diagnosis of HIV-associated TB [8], especially among those with advanced immunosuppression [9]–[12]. Patients testing LAM-positive have poor prognostic characteristics and clinical outcomes [12]–[14] and the ability to detect LAM appears to be associated with disease severity [15]. Assays for urine LAM may potentially be useful in the diagnostic algorithm as a rule-in test for HIV-associated TB and the evidence is due to be assessed by the World Health Organisation (WHO) in 2014.

Two formats of LAM detection assays are currently commercially available: an enzyme-linked immunosorbent assay (ELISA – Clearview TB-ELISA, Alere Inc, Waltham, MA, USA) and a simple, lateral flow, point-of-care assay Determine TB-LAM (Alere Inc.). Both assay formats have a qualitative binary (positive or negative) read-out. However, the LAM ELISA can also be used to provide a quantitative read-out expressed as the optical density (OD) at 450 nm [16]. Evaluations of LAM ELISA have shown very strong, correlations between the OD and concentration of purified LAM [16]. However, little is known about whether LAM quantification provides additional clinically useful information. We therefore undertook this retrospective analysis of LAM ELISA data from a cohort study in South Africa to determine the relationship between LAM OD and markers of HIV disease progression, other prognostic indices, mycobacterial burden, and mortality.

## Methods

The extremely high burden of TB among treatment-naïve patients at the antiretroviral treatment (ART) clinic in Gugulethu Township, Cape Town has been previously characterised in detail [17], [18]. Written informed consent was provided by all patients and the study was jointly approved by the ethics committees of the University of Cape Town, Cape Town, South Africa, and the London School of Hygiene & Tropical Medicine, London, UK.

Details of patient recruitment and laboratory procedures have previously been reported in parent studies [9], [19], [20]. Eligible patients were ART-naïve adults aged >18 years without a current TB diagnosis attending an ART centre in a Cape Town township for treatment initiation. All patients received trimethoprim-sulphamethoxazole prophylaxis.

Prospectively recruited patients had demographic details recorded and a standardized symptom-screening questionnaire completed prior to starting ART. Two sputum samples, one spot and one induced sample [21], were obtained from all patients. Additionally, all patients provided urine samples that were collected in sterile containers and stored at −20°C within 3 hours of collection. Venous blood was collected for measurement of CD4 cell counts and plasma viral load. We determined clinical outcomes of patients up to 90 days during routine follow-up within the ART service as previously described [13].

### Procedures

Laboratory procedures have been described in detail elsewhere [9], [19]. In brief, sputum samples were processed in an accredited laboratory and were decontaminated using N-acetyl-L-cysteine and sodium hydroxide. Sputum was tested using fluorescence microscopy and a ‘smear-positive’ result was defined by any smear graded as scanty, 1+, 2+ or 3+. Sputum was also tested by liquid culture using Mycobacterial Growth Indicator Tubes (MGIT, Becton Dickinson, Sparks, Maryland, USA).

Stored urine samples were defrosted and retrospectively analysed in duplicate for the presence of LAM using Clearview MTB-ELISA (Alere Inc.), with strict adherence to the manufacture's instructions. OD readings were used to quantitatively express urine LAM results. Each patient's final OD was determined by subtracting the mean negative control OD from the mean patient sample reading with a minimum value of 0. A positive LAM result was defined by an OD of at least 0.1 in accordance with the manufacturer's instructions. Defrosted urine samples (2.0 mL) were also retrospectively tested using Xpert MTB/RIF (Cepheid Inc, Sunnyvale, CA USA) according to manufacturer's instructions. C-reactive protein (CRP) concentrations were measured in serum samples using the Quantikine enzyme-linked immunosorbent assay (R&D Systems Inc., Minneapolis, MN, USA) per manufacturer's instructions.

### Definitions and analysis

A confirmed TB case was defined by a patient having *Mycobacterium tuberculosis* cultured from one or more sputum samples. Patient haemoglobin values were used to classify anaemia according to WHO criteria [22]. Patients were grouped into one of three mutually exclusive groups according to their urine LAM result and corresponding LAM OD where patients were urine LAM-negative, urine LAM-positive with a low OD (OD<0.50) or urine LAM-positive with a high OD (OD≥0.50) as this cut-off approximated to the median. Proportions were compared using either chi-squared tests or Fisher's exact tests as indicated and medians were compared using Kruskal-Wallis tests. The relationship between LAM ODs and indices of clinical prognosis, mycobacterial burden and mortality was examined using box and whisker plots and the corresponding medians were compared using either Wilcoxon rank-sum tests or Kruskal-Wallis tests where appropriate. Spearman rank correlation coefficients were used to test the relationship between LAM ODs and several variables.

## Results

Of 602 enrolled patients, 535 (88.9%) produced at least 1 sputum sample and 1 urine sample. Among those with both sputum culture and LAM OD results available (n = 514), 84 cases of sputum culture-positive TB were confirmed (prevalence 16.3% [95%CI, 13.2–19.8]). Those with TB were young and the majority was female (**Table 1**). The median CD4 cell count among these patients was 138 cells/µL (IQR, 63–205) and the median plasma viral load was 4.8 log copies/mL (IQR, 4.4–5.3).

**Table 1:** Characteristics of patients (n = 84) with confirmed tuberculosis (TB) stratified according to urine concentration of lipoarabinomannan (LAM) expressed as ELISA optical density (OD).

	All TB patients (n = 84)	TB Patients categorised by urine LAM result*			p-value
		LAM-negative (n = 61)	LAM-positive: low OD (n = 10)	LAM-positive: high OD (n = 13)	
**Patient characteristics**					
Age (years), median (IQR)	33.1 (28.4–40.2)	34.4 (29.1–42.4)	30.1 (23.1–33.6)	29.4 (25.8–33.4)	0.022
Female, no. (%)	51 (60.7)	33 (54.1)	8 (80.0)	10 (76.9)	0.171
BMI (kg/m^2^), median (IQR)	21.2 (19.2–26.0)	21.4 (20.0–26.0)	19.8 (16.8–25.9)	19.8 (17.1–22.2)	0.112
**Blood tests** a					
Haemoglobin (g/dL), median (IQR)	10.9 (8.8–12.4)	11.7 (10.3–13.0)	8.9 (7.9–9.9)	7.5 (7.0–8.4)	<0.001
C-reactive protein (mg/L), median (IQR)	54.1 (18.5–202.7)	37.9 (11.8–91.1)	125.7 (70.6–207.3)	207.4 (130.0)	<0.001
Absolute neutrophil count (x10^9^/L), median (IQR)	3.3 (2.4–5.0)	3.0 (2.0–4.0)	3.8 (2.5–5.0)	7.9 (4.4–11.5)	<0.001
**CD4 cell count (cells/µL)^b^**					
Median	138 (63–205)	176 (100–214)	83 (30–145)	37 (10–112)	<0.001
<50	18 (21.7)	7 (11.7)	4 (40.0)	7 (53.9)	0.010
50–99	11 (13.3)	8 (13.3)	2 (20.0)	1 (7.7)	
100–149	17 (20.5)	11 (18.3)	2 (20.0)	4 (30.8)	
150–199	13 (15.7)	12 (20.0)	0	1 (7.7)	
≥200	24 (28.9)	22 (36.7)	2 (20.0)	0	
HIV viral load (log copies/mL), median (IQR)	4.8 (4.4–5.3)	4.7 (4.2–5.1)	5.0 (4.7–5.5)	5.4 (5.2–5.6)	<0.001

a Blood test results available for 81 patients, ^b^CD4 cell counts available for 83 patients; *LAM-negative patients had an OD<0.10, LAM-positive: low OD patients had an OD ≥0.10 and <0.50, LAM-positive: high OD patients had an OD≥0.50.

### Characteristics of patients grouped by LAM result and corresponding OD

Among the 84 patients with HIV-associated TB, 23 (27.4%) tested urine LAM-positive and 61 (72.6%) were LAM-negative. The median OD among patients testing LAM-positive was 0.68 (IQR 0.16–2.43; range, 0.10–3.29). Of those with a positive LAM result, 10 had a low OD (<0.50) while 13 had a high OD (≥0.50). The characteristics of patients were explored and groups whose urine tested LAM-negative, LAM-positive (low OD) and LAM-positive (high OD) were compared (**Table 1**). Increasing LAM ODs across these groups were associated with lower CD4 cell counts and haemoglobin levels and higher HIV viral loads, CRP concentrations and blood neutrophil counts.

### Relationship between LAM OD and indices of HIV disease and clinical prognosis in patients with confirmed TB

**Figure 1:**
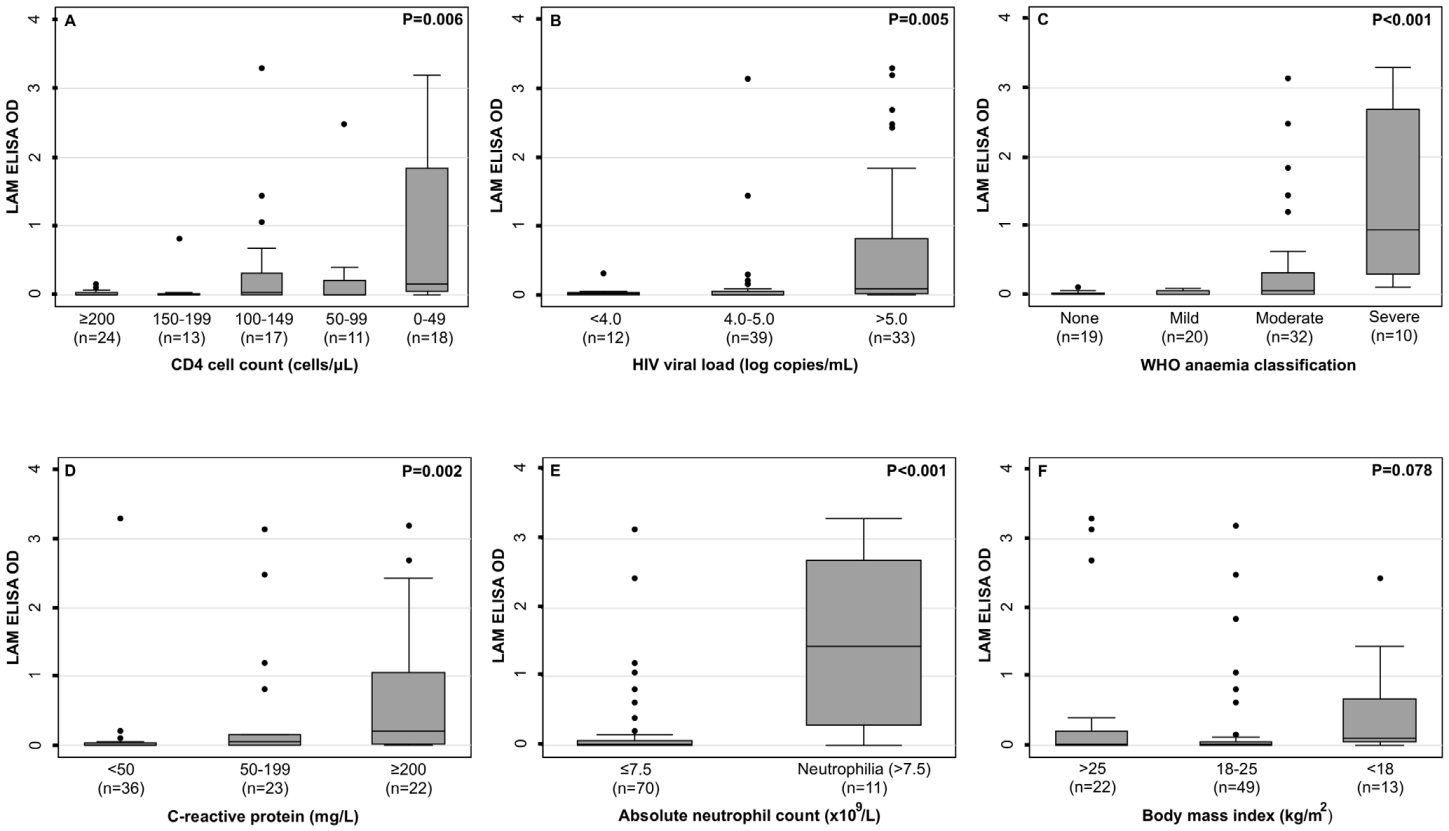
Box and whisker plot of urine concentrations of lipoarabinomannan (LAM) expressed as ELISA optical densities in patients with HIV-associated tuberculosis stratified by indices of HIV disease progression and clinical prognosis: a) CD4 cell count (n = 83), b) log HIV viral load (n = 84) c) WHO anaemia severity (n = 81), d) C-reactive protein concentration (n = 81), e) absolute neutrophil count (n = 81) and f) body mass index (n = 84). Bars, box and whiskers indicate medians, 25th and 75th centiles and ranges, respectively. P-values are either Wilcoxon rank-sum tests or Kruskal-Wallis for comparison of medians.

We further explored the relationship between LAM OD and markers of HIV disease progression and other prognostic indices among all TB patients (n = 84) (**Figure 1**). LAM ODs were substantially higher among those with the lowest CD4 counts (0–49 cells/µL) (**Figure 1a**) and the highest levels of HIV plasma viraemia (**Figure 1b**). LAM OD was also substantially higher among patients with severe anaemia (**Figure 1c**), the highest plasma CRP concentrations (≥200 mg/L) (**Figure 1d**) and in those with neutrophilia (>7.5×10^9^/L) (**Figure 1e**). No clear association was observed between LAM OD and body mass index (BMI) (**Figure 1f**).

### Factors correlated with LAM ODs in LAM-positive patients

Next we sought to determine which factors were correlated with LAM ODs when analysis was restricted to those with a positive LAM result and using continuous data. Since the data were not normally distributed or linearly correlated with any variables tested, non-parametric Spearman's rank correlation coefficients were used. Among 23 patients testing LAM-positive, absolute neutrophil count was the variable most strongly correlated with LAM OD (**Table 2**). Both haemoglobin levels and CD4 cell counts also demonstrated inverse correlations with LAM ODs but other variables were not correlated with LAM OD. Thus, LAM ODs correlated with more severe anaemia, CD4 lymphocytopenia and neutrophilia.

**Table 2:** Spearman's rank correlation coefficients for factors associated with urine lipoarabinomannan (LAM) ELISA optical densities among patients testing urine LAM positive (n = 23).

	Spearman's correlation coefficient	p-value
**Age, years**	0.020	0.929
**Female**	0.175	0.425
**Body mass index, kg/m^2^**	0.302	0.161
**Haemoglobin, g/dL**	−0.431	0.040
**C-reactive protein, mg/L**	0.209	0.340
**Absolute neutrophil count, ×10^9^/L**	0.599	0.003
**CD4 cell count, cells/µL**	−0.414	0.050
**HIV viral load, log copies/mL**	0.255	0.240

### Relationship between LAM OD and mycobacterial burden

**Figure 2:**
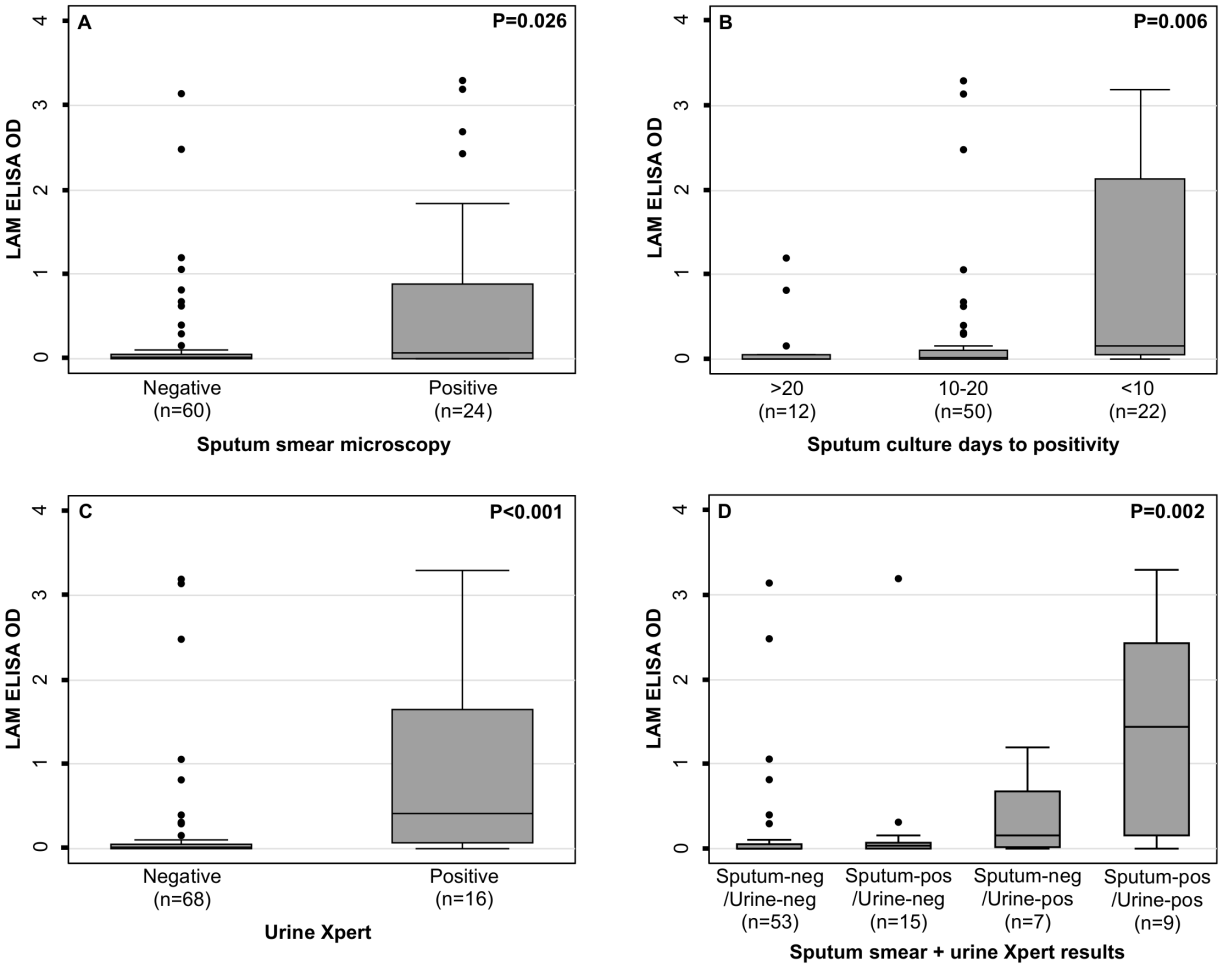
Box and whisker plot of urine concentrations of lipoarabinomannan (LAM) expressed as ELISA optical densities in patients with HIV-associated tuberculosis stratified by indices of mycobacterial burden: a) sputum smear microscopy result (n = 84), b) sputum culture days to positivity (n = 84), c) urine Xpert result (n = 84), d) a composite index combining sputum smear microscopy and urine Xpert results (pos  =  positive; neg  =  negative). Bars, box and whiskers indicate medians, 25th and 75th centiles and ranges, respectively. P-values are either Wilcoxon rank-sum tests or Kruskal-Wallis for comparison of medians.

Among all patients with confirmed TB (n = 84), LAM ODs were strongly associated with indices of mycobacterial burden in both sputum and urine (**Figure 2**). Higher LAM ODs were observed in patients with sputum smear-positive disease (**Figure 2a**) and in those with the shortest times to sputum culture-positivity (**Figure 2b**). LAM OD was also associated with detection of *M. tuberculosis* bacilli in urine using the Xpert MTB/RIF assay (**Figure 2c**). Combining sputum and urine results in a composite index of mycobacterial burden also revealed a striking positive association with LAM ODs (**Figure 2d**).

### Relationship between LAM OD and mortality

Finally we sought to determine if LAM OD was associated with mortality. Among 84 patients with confirmed TB, 5 (6%) patients died within 3 months of enrolment. The median LAM OD among patients who died was at least more than 6.8-fold higher than that of patients who remained alive at 3 months (0.68 [IQR, 0.62–2.43] versus <0.10, respectively; p = 0.002). Of patients testing urine LAM-positive with a high OD, 4 of 13 (31%) died compared to 1 of 10 (10%) patients testing urine LAM-positive with a low OD and none of the 61 (0%) patients who tested LAM-negative (Chi-squared for trend; P = 0.006). However, the small number of total deaths precluded assessment of comparative mortality risk in groups stratified by low and high LAM OD results.

## Discussion

In this study of HIV-infected patients with sputum culture-positive TB, we found that higher concentrations of urine LAM (as reflected by ELISA OD) were associated with extremely advanced HIV disease progression and higher mycobacterial burden. Furthermore, higher urine LAM concentrations were strongly associated with a range of poor prognostic indices that are known to be associated with increased mortality risk. Collectively, these results suggest that a quantitative or semi-quantitative read-out of urine LAM concentration may provide additional useful clinical information.

Urine LAM testing may have an important role in the diagnostic algorithm for HIV-associated TB. Although the overall sensitivity of this assay is low, it nevertheless has highest sensitivity among patients with advanced immunosuppression [9]–[12], [14] and moderate/severe anaemia [23]. Additionally, the availability of a rapid, point-of-care assay version makes urinary LAM testing much more user-friendly, permitting rapid initiation of TB treatment at a single clinic visit for a proportion of patients [8]. While low overall sensitivity means that urine LAM assays are inappropriate as stand-alone tests, high specificity make them useful add-on tests within the TB diagnostic algorithm to quickly ‘rule-in’ and confirm TB [24]. A growing evidence base indicates that a major strength of urine LAM testing is that LAM-positive disease correlates with poor prognosis [12], [13], [25]. Therefore, a key role for urinary LAM testing may be point-of-care diagnosis of TB in the very sickest patients who urgently require immediate anti-tuberculosis treatment. The present data further demonstrate that having higher urine LAM concentration is very strongly associated with adverse prognostic characteristics and might be used as an indicator for the need for adjunctive interventions, including more intensive treatment and follow-up.

High ELISA ODs were strongly associated with markers of very advanced HIV disease, and high mycobacterial burden as reflected by both sputum and urine assays. Since the Xpert MTB/RIF assay detects DNA in whole *Mycobacterium tuberculosis* bacilli, urine testing positive by this assay indicates renal involvement in patients with disseminated disease. It seems likely that renal involvement in those with disseminated TB is also the mechanism of LAM antigenuria. In support of this, it has been previously demonstrated that higher LAM concentrations were associated with mycobacteremia and were highest in those with proven multi-compartmental disease [26]. Post-mortem studies conducted throughout sub-Saharan Africa and India have shown that between 38% and 67% of medical in-patients with HIV/AIDS had evidence of TB at the time of death that was associated with very high bacillary loads and widely disseminated disease with multi-organ involvement [27]–[32]. Of note, several of these studies reported that renal involvement with TB was extremely common [28], [29], [32].

While LAM ODs were associated with several indices of poor prognosis, the strongest correlation was with blood neutrophil counts and the highest median LAM OD was seen in patients with neutrophilia. Neutrophilia is directly associated with higher mycobacterial burden [33] and is an independent predictor of mortality in patients with TB [34]. It is not clear whether the high neutrophil counts observed in LAM-positive patients reflect high mycobacterial load alone or are indicative of concurrent sepsis in a proportion of these patients who have such advanced immunosuppression that they are at high risk for bacterial sepsis. Thus, high LAM concentrations may potentially be indicative of a need for adjunctive antibiotics in addition to anti-tuberculosis treatment and trimethoprim-sulphamethoxazole prophylaxis.

Very high LAM ODs were seen in those with severe anaemia and in those with greatly elevated CRP levels. Both of these parameters are likely to be associated with high mycobacterial burden and disseminated disease and are known to be associated with mortality risk [23], [35]. Patients who died within 3 months of enrolment had much higher urine LAM concentrations than those who survived. Four of the 5 patients who died had high LAM ODs (>0.5). However, the small total number of deaths in this study precluded assessment of comparative mortality risk of patients testing urine LAM-positive with a low OD or a high OD. These results suggest that not only is LAM-positivity predictive of very poor clinical prognosis, but also that a LAM assay with a semi-quantitative or quantitative read out may provide further prognostic information. A larger study with a greater number of deaths is needed to accurately define the relationship between urine LAM concentration and mortality.

Determine TB-LAM is a low-cost, rapid, point-of-care assay that has similar sensitivity and specificity to the laboratory-based LAM ELISA for HIV-associated TB [9]. Although it is marketed for use as a qualitative (positive/negative) assay, the read-out displays a band of variable intensity that can be graded by comparison to the included manufacturer's reference card. This might therefore provide a semi-quantitative result although use of the assay in this way has not been validated. Increasing grade of Determine-TB LAM may be directly correlated with higher LAM ODs and poorer prognosis although these relationships remain to be defined in future studies. It may be useful clinically if future versions of this assay have two positive bands: one to indicate a positive result with a low LAM concentration and another to indicate a positive result with a high LAM concentration.

Study strengths include a well-characterised, unselected patient cohort that was systematically screened for TB regardless of clinical presentation as well as the use of liquid culture as a reference standard for TB diagnosis. A range of prognostic indices were examined and mortality risk was prospectively determined. This study had some limitations. All patients had sputum culture-positive disease and patients with isolated extrapulmonary disease would not have been included. We quantified LAM in terms of ELISA OD but did not determine absolute concentrations in urine. Additionally, in the parent study, the Determine TB-LAM assay read-out was qualitatively recorded as either a positive or negative result and so the relationship between LAM ELISA OD and the Determine TB-LAM POC assay positivity grade could not be examined. Finally, due a small number of deaths occurring among patients with confirmed HIV-associated TB, we were unable to undertake a multivariable logistic regression analysis to determine if urine LAM OD was independently associated with mortality. Future studies among in-patients in whom mortality risk is much higher may help to define this relationship further.

In conclusion, among patients with HIV-associated TB, urine LAM is quantitatively associated with advanced HIV disease progression, higher mycobacterial burden and poorer prognostic characteristics that are known to be associated with increased mortality risk. Urine LAM assays with a semi-quantitative or quantitative read out may help to identify those with the poorest prognosis and requiring adjunctive interventions. Such an assay may therefore have increased clinical utility.

## References

[1] World Health Organization (2013) Global tuberculosis report 2013. World Health Organization, Geneva, 2013. Accessible at the following URL: http://appswhoint/iris/bitstream/10665/91355/1/9789241564656_engpdf.

[2] Lawn SD, Wood R (2011) Tuberculosis in Antiretroviral Treatment Services in Resource-Limited Settings: Addressing the Challenges of Screening and Diagnosis. J Infect Dis 204: S1159–S1167 10.1093/infdis/jir411 21996698 PMC3192543

[3] Getahun H, Harrington M, O'Brien R, Nunn P (2007) Diagnosis of smear-negative pulmonary tuberculosis in people with HIV infection or AIDS in resource-constrained settings: informing urgent policy changes. Lancet 369: 2042–2049 10.1016/S0140-6736(07)60284-0 17574096

[4] Dawson R, Masuka P, Edwards DJ, Bateman ED, Bekker L-G, et al (2010) Chest radiograph reading and recording system: evaluation for tuberculosis screening in patients with advanced HIV. Int J Tuberc Lung Dis 14: 52–58. 20003695 PMC3647461

[5] Rylance J, Pai M, Lienhardt C, Garner P (2010) Priorities for tuberculosis research: a systematic review. Lancet Infect Dis 10: 886–892 10.1016/S1473-3099(10)70201-2 21050822 PMC2992175

[6] Zumla A, Kim P, Maeurer M, Schito M (2013) Zero deaths from tuberculosis: progress, reality, and hope. Lancet Infect Dis 13: 285–7 10.1016/S1473-3099(13)70039-2 23531385

[7] Lawn SD, Mwaba P, Bates M, Piatek A, Alexander H, et al (2013) Advances in tuberculosis diagnostics: the Xpert MTB/RIF assay and future prospects for a point-of-care test. Lancet Infect Dis 13: 349–361 10.1016/S1473-3099(13)70008-2 23531388 PMC4844338

[8] Lawn SD (2012) Point-of-care detection of lipoarabinomannan (LAM) in urine for diagnosis of HIV-associated tuberculosis: a state of the art review. BMC Infect Dis 12: 103 10.1186/1471-2334-12-103 22536883 PMC3423001

[9] Lawn SD, Kerkhoff AD, Vogt M, Wood R (2012) Diagnostic accuracy of a low-cost, urine antigen, point-of-care screening assay for HIV-associated pulmonary tuberculosis before antiretroviral therapy: a descriptive study. Lancet Infect Dis 12: 201–209 10.1016/S1473-3099(11)70251-1 22015305 PMC3315025

[10] Peter JG, Theron G, van Zyl-Smit R, Haripersad A, Mottay L, et al (2012) Diagnostic accuracy of a urine lipoarabinomannan strip-test for TB detection in HIV-infected hospitalised patients. **Eur Respir** J 40: 1211–1220 10.1183/09031936.00201711 22362849 PMC5523653

[11] Lawn SD, Edwards DJ, Kranzer K, Vogt M, Bekker L-G, et al (2009) Urine lipoarabinomannan assay for tuberculosis screening before antiretroviral therapy diagnostic yield and association with immune reconstitution disease. AIDS 23: 1875–1880. 20108382 10.1097/qad.0b013e32832e05c8

[12] Shah M, Variava E, Holmes CB, Coppin A, Golub JE, et al (2009) Diagnostic accuracy of a urine lipoarabinomannan test for tuberculosis in hospitalized patients in a High HIV prevalence setting. J Acquir Immune Defic Syndr 52: 145–151 10.1097/QAI.0b013e3181b98430 19692904 PMC2815254

[13] Lawn SD, Kerkhoff AD, Vogt M, Wood R (2012) Clinical significance of lipoarabinomannan detection in urine using a low-cost point-of-care diagnostic assay for HIV-associated tuberculosis. AIDS 26: 1635–1643 10.1097/QAD.0b013e3283553685 22555166

[14] Gounder CR, Kufa T, Wada NI, Mngomezulu V, Charalambous S, et al (2011) Diagnostic accuracy of a urine lipoarabinomannan enzyme-linked immunosorbent assay for screening ambulatory HIV-infected persons for tuberculosis. J Acquir Immune Defic Syndr 58: 219–223 10.1097/QAI.0b013e31822b75d4 21765364 PMC3175281

[15] Lawn SD, Kerkhoff AD, Vogt M, Wood R (2013) HIV-associated tuberculosis: relationship between disease severity and the sensitivity of new sputum-based and urine-based diagnostic assays. BMC Med 11: 231 10.1186/1741-7015-11-231 24168211 PMC4231603

[16] Boehme C, Molokova E, Minja F, Geis S, Loscher T, et al (2005) Detection of mycobacterial lipoarabinomannan with an antigen-capture ELISA in unprocessed urine of Tanzanian patients with suspected tuberculosis. Trans R Soc Trop Med Hyg 99: 893–900 10.1016/j.trstmh.2005.04.014 16139316

[17] Lawn SD, Myer L, Bekker L-G, Wood R (2006) Burden of tuberculosis in an antiretroviral treatment programme in sub-Saharan Africa: impact on treatment outcomes and implications for tuberculosis control. AIDS 20: 1605–1612 10.1097/01.aids.0000238406.93249.cd 16868441

[18] Lawn SD, Myer L, Orrell C, Bekker L-G, Wood R (2005) Early mortality among adults accessing a community-based antiretroviral service in South Africa: implications for programme design. AIDS 19: 2141–2148. 16284464 10.1097/01.aids.0000194802.89540.e1

[19] Lawn SD, Brooks SV, Kranzer K, Nicol MP, Whitelaw A, et al (2011) Screening for HIV-associated tuberculosis and rifampicin resistance before antiretroviral therapy using the Xpert MTB/RIF assay: a prospective study. PLoS Med 8: e1001067 10.1371/journal.pmed.1001067 21818180 PMC3144215

[20] Lawn SD, Kerkhoff AD, Vogt M, Ghebrekristos Y, Whitelaw A, et al (2012) Characteristics and early outcomes of patients with Xpert MTB/RIF-negative pulmonary tuberculosis diagnosed during screening before antiretroviral therapy. Clin Infect Dis 54: 1071–1079 10.1093/cid/cir1039 22318975 PMC3309885

[21] Lawn SD, Kerkhoff AD, Pahlana P, Vogt M, Wood RR (2012) Diagnostic yield of tuberculosis using sputum induction in HIV-positive patients before antiretroviral therapy. Int J Tuberc Lung Dis 16: 1354–1357 10.5588/ijtld.12.0174 22862896

[22] World Health Organization (2011) Haemoglobin Concentrations for the Diagnosis of Anaemia and Assessment of Severity. Mineral Nutrition Information System. Geneva, Switzerland: World Health Organization. Available at: http://www.who.int/vmnis/indicators/haemoglobin.pdf. 2011.

[23] Kerkhoff AD, Wood R, Vogt M, Lawn SD (2013) Predictive value of anemia for tuberculosis in HIV-infected patients in sub-Saharan Africa: an indication for routine microbiological investigation using new rapid assays. J Acquir Immune Defic Syndr. doi:10.1097/QAI.0000000000000091 10.1097/QAI.0000000000000091 PMC3981888 24346639

[24] Lawn SD, Dheda K, Kerkhoff AD, Peter JG, Dorman S, et al (2013) Determine TB-LAM lateral flow urine antigen assay for HIV-associated tuberculosis: recommendations on the design and reporting of clinical studies. BMC Infect Dis 13: 407 10.1186/1471-2334-13-407 24004840 PMC3846798

[25] Talbot E, Munseri P, Teixeira P, Matee M, Bakari M, et al (2012) Test Characteristics of Urinary Lipoarabinomannan and Predictors of Mortality among Hospitalized HIV-Infected Tuberculosis Suspects in Tanzania. PLoS ONE 7: e32876 10.1371/journal.pone.0032876.t004 22412939 PMC3297608

[26] Shah M, Martinson NA, Chaisson RE, Martin DJ, Variava E, et al (2010) Quantitative Analysis of a Urine-Based Assay for Detection of Lipoarabinomannan in Patients with Tuberculosis. J Clin Micro 48: 2972–2974 10.1128/JCM.00363-10 PMC2916614 20534796

[27] Lucas SB, Hounnou A, Peacock C, Beaumel A, Djomand G, et al (1993) The mortality and pathology of HIV infection in a west African city. AIDS 7: 1569–1579. 7904450 10.1097/00002030-199312000-00005

[28] Rana FS, Hawken MP, Mwachari C, Bhatt SM, Abdullah F, et al (2000) Autopsy study of HIV-1-positive and HIV-1-negative adult medical patients in Nairobi, Kenya. J Acquir Immune Defic Syndr 24: 23–29. 10877491 10.1097/00126334-200005010-00004

[29] Ansari NA, Kombe AH, Kenyon TA, Hone NM, Tappero JW, et al (2002) Pathology and causes of death in a group of 128 predominantly HIV-positive patients in Botswana, 1997-1998. Int J Tuberc Lung Dis 6: 55–63. 11931402

[30] Cohen T, Murray M, Wallengren K, Alvarez GG, Samuel EY, et al (2010) The Prevalence and Drug Sensitivity of Tuberculosis among Patients Dying in Hospital in KwaZulu-Natal, South Africa: A Postmortem Study. PLoS Med 7: e1000296 10.1371/journal.pmed.1000296.t005 20582324 PMC2889914

[31] Wong EB, Omar T, Setlhako GJ, Osih R, Feldman C, et al (2012) Causes of Death on Antiretroviral Therapy: A Post-Mortem Study from South Africa. PLoS ONE 7: e47542 10.1371/journal.pone.0047542.t004 23094059 PMC3472995

[32] Lanjewar DN, Duggal R (2001) Pulmonary pathology in patients with AIDS: an autopsy study from Mumbai. HIV Med 2: 266–271. 11737408 10.1046/j.1468-1293.2001.00079.x

[33] Kerkhoff AD, Wood R, Lowe DM, Vogt M, Lawn SD (2013) Blood neutrophil counts in HIV-infected patients with pulmonary tuberculosis: association with sputum mycobacterial load. PLoS ONE 8: e67956 10.1371/journal.pone.0067956 23874476 PMC3706476

[34] Lowe DM, Bandara AK, Packe GE, Barker RD, Wilkinson RJ, et al (2013) Neutrophilia independently predicts death in tuberculosis. Eur Resp J 42: 1752–1757 10.1183/09031936.00140913 PMC4176760 24114967

[35] Lawn SD, Kerkhoff AD, Vogt M, Wood RR (2013) Diagnostic and prognostic value of serum C-reactive protein for screening for HIV-associated tuberculosis. Int J Tuberc Lung Dis 17: 636–643 10.5588/ijtld.12.0811 23575330 PMC3816250

